# Alpha‐synuclein at the nexus of genes and environment: the impact of environmental enrichment and stress on brain health and disease

**DOI:** 10.1111/jnc.14787

**Published:** 2019-07-09

**Authors:** Zinah Wassouf, Julia M. Schulze‐Hentrich

**Affiliations:** ^1^ German Center for Neurodegenerative Diseases Göttingen Germany; ^2^ Institute of Medical Genetics and Applied Genomics University of Tübingen Tübingen Germany; ^3^ Present address: German Center for Neurodegenerative Diseases Göttingen Germany

**Keywords:** alpha‐synuclein, enriched environment, epigenetics, neurodegeneration, Parkinson’s disease, stress

## Abstract

Accumulation of alpha‐synuclein protein aggregates is the hallmark neuropathologic feature of synucleinopathies such as Parkinson’s disease. Rare point mutations and multiplications in *SNCA*, the gene encoding alpha‐synuclein, as well as other genetic alterations are linked to familial Parkinson’s disease cases with high penetrance and hence constitute major genetic risk factors for Parkinson’s disease. However, the preponderance of cases seems sporadic, most likely based on a complex interplay between genetic predispositions, aging processes and environmental influences. Deciphering the impact of these environmental factors and their interactions with the individual genetic background in humans is challenging and often requires large cohorts, complicated study designs, and longitudinal set‐ups. In contrast, rodent models offer an ideal system to study the influence of individual environmental aspects under controlled genetic background and standardized conditions. In this review, we highlight findings from studies examining effects of environmental enrichment mimicking stimulation of the brain by its physical and social surroundings as well as of environmental stressors on brain health in the context of Parkinson’s disease. We discuss possible internal molecular transducers of such environmental cues in Parkinson’s disease rodent models and emphasize their potential in developing novel avenues to much‐needed therapies for this still incurable disease.

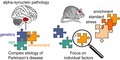

This article is part of the Special Issue “Synuclein”

**Abbreviations used**
6‐OHDA: 6‐hydroxydopamine
AD: Alzheimer’s disease
α-syn: alpha‐synuclein
BDNF: brain‐derived neurotrophic factor
EE: environmental enrichment
EHMT2: euchromatic histone‐lysine N‐methyltransferase 2
GCs: glucocorticoids
GR: glucocorticoid receptor
HD: Huntington’s disease
IGF‐1: insulin‐like growth factor‐1
MPTP: 1‐methyl‐4‐phenyl‐1,2,3,6‐tetrahydropyridine
NGF: nerve growth factor
PD: Parkinson’s disease
RE1: restrictive element 1
REST: restrictive element 1‑silencing transcription factor
SNAP25: synaptosomal‐associated protein
VEGF: vascular endothelial growth factor

Alpha‐synuclein (α‐syn) is a key presynaptic protein in Parkinson’s disease (PD) and other synucleinopathies. Misfolding and aggregation of α‐syn is a central event in synucleinopathies, and the presence of eosinophilic intracellular inclusions that contain abundant levels of α‐syn reflects the main neuropathological hallmark of these diseases, known as Lewy pathology (Spillantini *et al.,* 1997; Spillantini *et al.,* 1998; Shults, 2006). Yet, the factors triggering α‐syn pathology and the resulting cellular toxicity are still largely enigmatic.

## The physiological function of alpha‐synuclein

α‐syn belongs to the synuclein family of proteins together with its close homologs beta‐ and gamma‐synuclein. Synuclein proteins are prevalently expressed in the nervous system (Jakes *et al.,* 1994; Lavedan *et al.,* 1998a; Lavedan *et al.,* 1998b) and localize preferentially to presynaptic terminals (Jakes *et al.,* 1994; George, 2002; Ninkina *et al.,* 2012). Despite its ubiquitous neuronal expression, α‐syn has also been detected in peripheral tissues and blood cells (Askanas *et al.,* 2000; Shin *et al.,* 2000; Ltic *et al.,* 2004; Nakai *et al.,* 2007). In neuronal cells, the apparent nuclear and synaptic localization of α‐syn suggests a physiological function in both compartments. Despite some controversy about α‐syn’s nuclear localization, current reports support the existence of α‐syn in the nucleus and its potential direct interaction with DNA (Goers *et al.,* 2003; Ma *et al.,* 2014b; Pinho *et al.,* 2019). In addition, nuclear α‐syn has been detected in transgenic models such as mice (Masliah *et al.,* 2000; Goers *et al.,* 2003), Drosophila (Takahashi *et al.,* 2003), and cells (McLean *et al.,* 2000; Specht *et al.,* 2005), as well as in human brain samples (Siddiqui *et al.,* 2012). Apparently, the abnormal accumulation of α‐syn in the nucleus is associated with DNA damage and neurotoxicity (Kontopoulos *et al.,* 2006; Padmaraju *et al.,* 2011; Ma *et al.,* 2014a). Besides the potential binding of α‐syn to DNA and direct regulation of gene expression under specific conditions (Martins *et al.,* 2011; Siddiqui *et al.,* 2012), α‐syn may also interfere with epigenetic processes regulating gene expression (Kontopoulos *et al.,* 2006; Desplats *et al.,* 2011). These processes do not necessarily require the physical presence of α‐syn inside the nucleus as their regulation may also rely on a number of cellular mediators bridging the effects of α‐syn to the nucleus (Jin *et al.,* 2011).

The high concentration of α‐syn in presynaptic terminals, its association with synaptic vesicles (Maroteaux *et al.,* 1988; Kahle *et al.,* 2000), interaction with presynaptic proteins and function as a SNARE chaperon essential for vesicle fusion (Burre *et al.,* 2010; Chen *et al.,* 2013; Zaltieri *et al.,* 2015) suggest a role of α‐syn in regulating synaptic homeostasis and neurotransmitters release including dopamine. While knockout models for α‐syn exhibit normal basal hippocampal synaptic transmission, significant impairments in hippocampal synaptic responses are evident under conditions capable of exhausting docked as well as reserve pool vesicles (Cabin *et al.,* 2002). A slower refilling of the docked pools from the reserve pool, indicates a role for endogenous α‐syn in synaptic vesicles trafficking and maintenance (Abeliovich *et al.,* 2000; Cabin *et al.,* 2002). A reduction in the distal pool of synaptic vesicles upon α‐syn antisense oligonucleotide treatment in primary hippocampal neurons supports the capacity of α‐syn to regulate the vesicle pool size at synaptic terminals (Murphy *et al.,* 2000). Moreover, synuclein knockout mice show decreased SNARE‐complex assembly (Burre *et al.,* 2010) and changes in synaptic structure and size (Greten‐Harrison *et al.,* 2010) together with impairments in learning and memory tasks (Kokhan *et al.,* 2011; Kokhan *et al.,* 2012). These reports indicate that synucleins may not be vital to the basic machinery of synaptic transmission but rather contribute to the long‐term maintenance of synaptic functions (Chandra *et al.,* 2004).

The effect of α‐syn on the synaptic release machinery has been further investigated in α‐syn overexpressing models, where the consequent functional deficits based on α‐syn excess can be related to either toxic loss or gain of function of this protein (Benskey *et al.,* 2016; Collier *et al.,* 2016). α‐syn overexpression inhibits neurotransmitter release possibly due to impairments in synaptic vesicle recycling (Nemani *et al.,* 2010) and alterations in presynaptic proteins linked to exocytosis and endocytosis processes (Scott *et al.,* 2010). Moreover, overexpression of wildtype α‐syn, but not the A30P mutant, results in a decrease of dopamine release that correlates with a decreased density of dopaminergic vesicles (Gaugler *et al.,* 2012). Data from this report further suggest that the affinity of α‐syn protein for membranes plays an important role in the observed presynaptic pathology (Gaugler *et al.,* 2012). A reduction in dopamine reuptake and a dysfunction of the dopamine transporter in dopaminergic neurons have also been observed upon wildtype α‐syn overexpression in rats (Lundblad *et al.,* 2012). Altogether, despite the progress made in revealing the physiological function of α‐syn using knockout and overexpression models, many questions remain unanswered and require further research. A deeper insight into aspects of α‐syn functions and cellular distribution will aid in understanding the contribution of this protein under pathological conditions.

## The role of alpha‐synuclein in Parkinson’s disease

Traditionally described as the most common synucleinopathy and neurodegenerative movement disorder, PD is estimated to affect 2–3% of the population aged 65 years and above and its prevalence continues to increase with advancing age (Pringsheim *et al.,* 2014; Poewe *et al.,* 2017). A progressive degeneration of dopaminergic neurons in the substantia nigra pars compacta and depletion of striatal dopamine drive the manifestation of motor symptoms, such as bradykinesia, muscle rigidity, and tremor. Besides cardinal motor symptoms, a plethora of non‐motor and neuropsychiatric features accompany the disease from prodromal to advanced stages, likely involving additional brain circuitries besides the dopaminergic system (Goldman and Postuma, 2014; Poewe *et al.,* 2017; Przedborski, 2017). The characteristic Lewy pathology of both familial and sporadic forms of PD, is thought to precede the occurrence of motor symptoms and starts in the prodromal phase (Spillantini *et al.,* 1997; Spillantini *et al.,* 1998; Shults, 2006).

On a genetic level, point mutations in *SNCA* (A30P, E46K, H50Q, G51N, A53E, and A53T) are linked to rare familial cases of PD (Polymeropoulos *et al.,* 1997; Kruger *et al.,* 1998; Zarranz *et al.,* 2004; Lesage *et al.,* 2013; Proukakis *et al.,* 2013; Pasanen *et al.,* 2014). In addition, duplication and triplication of this locus cause familial PD in a gene dose‐dependent manner with early onset, varying pathology and clinical features (Singleton *et al.,* 2003; Chartier‐Harlin *et al.,* 2004; Ibanez *et al.,* 2004). Further, increased susceptibility to sporadic PD is associated with several genetic variants in the *SNCA* gene (Satake *et al.,* 2009; Simon‐Sanchez *et al.,* 2009). Similarly, genetic variants in *LRRK2*, *MAPT*, and *VPS13C* genes, also linked to familial cases of PD, are associated with increased risk for sporadic forms (Satake *et al.,* 2009; Simon‐Sanchez *et al.,* 2009; Nalls *et al.,* 2014). Despite the high disease penetrance of familial cases, clear monogenetic families are very rare, and the relative population risk based on genetic alterations alone seems to be limited to around 10% (Lesage and Brice, 2009). Thus, the great majority of PD patients do not have a straightforward genetic *SNCA* predisposition. However, all PD patients have α‐syn neuropathology, which in fact is the defining neuropathological hallmark needed for the definite *post‐mortem* diagnosis of PD.

## Environmental influences in Parkinson’s disease

As the majority of PD cases are sporadic with yet unknown etiology, the risk to develop PD seemingly arises from a complex interplay of genetic predispositions, aging processes, and environmental factors, resulting in a combined effect of both genetic and environmental elements that ultimately determine an individual’s susceptibility to the disease. In line with this idea, epidemiological evidence points towards a strong environmental component in disease etiology (Ascherio and Schwarzschild, 2016; Bellou *et al.,* 2016). Studies involving monozygotic or dizygotic twins, moreover, point at the relevant contribution of genetic and environmental factors. There, too, the evidence implies a strong environmental influence (Wirdefeldt *et al.,* 2004) as similar concordance rates for monozygotic and dizygotic twins have been observed (Tanner *et al.,* 1999). Accumulating evidence points at several environmental agents that potentially modify the risk of developing the disease. Most prominently, an increased risk for PD is associated with exposure to pesticides, herbicides, and insecticides (Brown *et al.,* 2005). Although debatable, living in a rural environment and drinking well water has been associated with a higher risk for PD, possibly due to the exposure to pesticides or other contaminants (Brown *et al.,* 2005; Ascherio and Schwarzschild, 2016). Furthermore, there is growing evidence for the role of heavy metals and exposure to organic solvents as putative risk factors for PD and Parkinsonism (Smargiassi *et al.,* 1998; Racette *et al.,* 2001; Goldman *et al.,* 2012; Racette, 2014). Besides the impact of environmental chemicals, clinical evidence indicates a role for environmental stress in PD onset (Zou *et al.,* 2013) supported by views on an analogy between stress‐induced pathological consequences and the neuronal deterioration observed in PD (Smith *et al.,* 2002). In contrast, seemingly lowering the risk for PD, coffee consumption, smoking, higher serum urate levels, and physical activity are inversely associated with PD (Hernan *et al.,* 2002; Ascherio and Schwarzschild, 2016). It is important to point out that in this context, the causality of these protective effects is highly debated, mainly because of the paucity of well‐designed studies in humans. Nevertheless, compelling evidence supports protective potentials of physical activity owing to highly significant associations with a reduced risk of developing PD (Bellou *et al.,* 2016). Taken together, an individual’s susceptibility to PD is likely influenced by a combination of life style factors and environmental exposures that act simultaneously or in sequence and interact with the individual’s genetic makeup rendering the organism more vulnerable to deleterious processes and neuronal attrition with advancing age (Carvey *et al.,* 2006; Sulzer, 2007).

## Epigenetics and alpha‐synuclein (patho‐)physiology

Owing to the dynamic nature of the epigenome and the susceptibility of the epigenetic landscape to the environment (Ost *et al.,* 2014; Allis and Jenuwein, 2016), epigenetic adaptations driven by environmental influences may hold the key to understanding the impact of the latter in health and disease. Changes in the epigenome have been linked to α‐syn and been related to several neurodegenerative disorders (Feng *et al.,* 2015; Landgrave‐Gomez *et al.,* 2015; Hwang *et al.,* 2017; Pavlou *et al.,* 2017). Evaluating genome‐wide DNA methylation profiles, differential methylation has been observed both in blood and brain tissue from PD patients (Masliah *et al.,* 2013). On bulk level, α‐syn has been proposed to alter DNA methylation by sequestering the maintenance DNA methyltransferase (Dnmt1) to the cytoplasm (Desplats *et al.,* 2011). Consistently, global DNA hypomethylation is accompanied by reduction of nuclear DNA methyltransferase 1 in human brain samples of patients with PD or dementia with Lewy bodies (Desplats *et al.,* 2011). Furthermore, α‐syn seemingly interacts with histones, first described in the nuclei of nigral neurons from toxin‐treated mice (Goers *et al.,* 2003). Such interactions between α‐syn and histones have been proposed to be toxic to neurons due to reduced histone acetylation, as rescue was observed when treating with histone deacetylase inhibitors (HDACis) in both cell culture and transgenic flies (Kontopoulos *et al.,* 2006). In addition, several transcription factors coordinating epigenetic regulation of genes essential for neuronal function have been associated with chromatin remodeling. Among such transcriptional regulators is the restrictive element 1‑silencing transcription factor (REST; also known as neuron‐restrictive silencer factor, NRSF), which acts via epigenetic mechanisms to instruct the expression of genes involved in neuronal function and survival (Noh *et al.,* 2012). REST’s role in maintaining normal brain development and function (Ballas and Mandel, 2005; Ooi and Wood, 2007; Baldelli and Meldolesi, 2015) implies that a dysregulation to this system might contribute to brain disorders and neurodegeneration. Indeed, REST seems dysregulated in the striatal tissue of Huntington’s disease (HD) patients and HD mouse models (Zuccato *et al.,* 2003), where nuclear availability of REST in striatal neurons is modulated through its interaction with the huntingtin protein. Recent studies have further highlighted the essential role of REST in healthy aging and implicated that dysregulation of REST is associated with cognitive decline and AD (Lu *et al.,* 2014). In the context of PD, 1‐methyl‐4‐phenyl‐1,2,3,6‐tetrahydropyridine (MPTP) treatment evokes REST expression and changes its subcellular distribution in dopaminergic cells (Yu *et al.,* 2009). In turn, REST depletion exacerbates the detrimental effect of MPTP treatment in mice implying a potential protective role for REST in neurons of the substantia nigra (Yu *et al.,* 2013). REST acts to repress gene expression by binding to restrictive element 1 (RE1) within the promoters of target genes. It recruits co‐repressors and promotes epigenetic remodeling to initiate gene silencing through a number of mechanisms such as recruiting histone deacetylases, histone methyltransferases, and histone demethylases (Huang *et al.,* 1999; Zhang *et al.,* 2002; Lee *et al.,* 2005; Kazantsev and Thompson, 2008). In a transgenic α‐syn Drosophila model and inducible SH‐SY5Y neuroblastoma cells, α‐syn overexpression increases levels of histone marks associated with heterochromatin, together with levels of EHMT2 (euchromatic histone‐lysine N‐methyltransferase 2, also known as G9a) that acts to modify H3K9 (Sugeno *et al.,* 2016). Indeed, a reduction of dimethylated H3K9 has been observed after EHMT2 inhibition in α‐syn expressing cells. Moreover, REST and H3K9 dimethylation are significantly enriched at RE1 sites of the synaptosomal‐associated protein (SNAP25), and their occupancy is further increased after α‐syn induction. This effect is accompanied by reduced SNAP25 expression consistent with the repressive function of H3K9me2 and the REST complex.

In conclusion, disturbances of epigenetic marks in PD likely play a role in disease unfolding and manifestation. Environment‐dependent epigenetic adaptations that impact the underlying gene expression are appealing regulatory layers that might harbor clues to the strong environmental component in PD. The reversibility of such epigenetic events by targeted therapies and environmental interventions offers great potential for new therapeutic strategies for this yet incurable disease.

## Using animal models to study environmental impacts

Studying the role of environmental exposures and their interaction with the individual's genetic background in humans is challenging and often requires large cohorts, complicated study designs, and long follow‐ups (years or even decades). In addition, many studies suffer from lack of consistency and specificity, possibly due to our highly complex living conditions that include a vast number of environmental variables and stimuli of different quality and quantity. As an alternative strategy, studying the impact of environmental modulation in disease models offers advantages with respect to I. Control and standardization of range and duration of environmental impacts. II. Focus on a specific stimulus on behavioral, structural, molecular, and functional levels. III. Trace the interaction between specific genetic features and environmental exposures.

In particular, rodent models are suitable to decipher the impact of environmental factors in PD as toxin‐induced as well as genetic models mimic the disease. Given the central role for α‐syn in PD pathogenesis, α‐syn transgenic models have emerged as valuable models in PD research and are well suited to study the role of environmental factors in disease unfolding. In this work, we highlight findings from studies examining environmental interventions in PD rodent models and point out how they relate to observations in PD patients. Specifically, we will focus on environmental enrichment, as a factor with protective impact on PD, and environmental stress, as a factor proposed to increase the risk of PD, and discuss the possible internal molecular transducers of such environmental influences in PD.

## Physical activity and environmental enrichment

**Figure 1:**
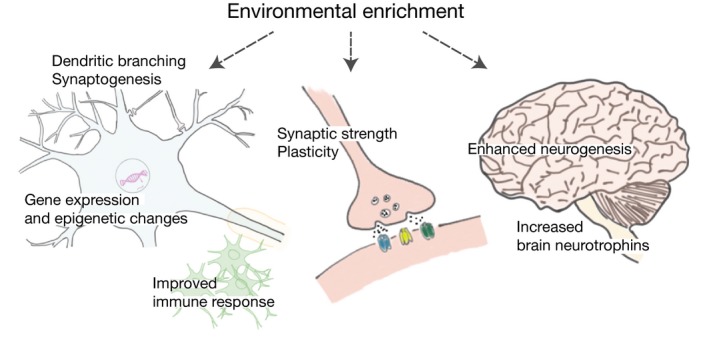
Environmental enrichment induces structural and functional modulations in the brain. The benefits of environmental enrichment (EE) include increased production and maturation of new neurons, enhanced levels of brain growth factors, synaptic strength, and plasticity. In addition, EE improves the immune condition of the brain, modulates gene expression, elicits epigenetic changes, and augments dendritic branching and synaptogenesis.

Environmental enrichment (EE), a term that has been coined for an enhanced motor and cognitive stimulation, is known to have a broad impact on the organism acting both peripherally and centrally. In the brain, the benefits of such an approach extend to several areas and involve structural and functional modulations that affect brain performance and resilience (Nithianantharajah and Hannan, 2006) (Fig. 1). Mimicking EE in laboratory rodents is achieved by placing objects, varying in shape, size, color, and texture, as well as running wheels in housing cages to enable more physical activity (Nithianantharajah and Hannan, 2006). The cages are typically larger in the enriched condition compared to standard housing, offering the possibility to house a higher number of animals in one cage resulting in enhanced social interactions. In addition, an EE also aims at greater cognitive stimulation and requires constant novel inputs for the animals achieved by regularly changing and rearranging objects in the EE cages.

As one of the best studied brain regions in this context, the hippocampus represents a central brain hub for integrating sensory information from external stimuli (Kempermann *et al.,* 1997). In the hippocampus, EE increases production and maturation of new neurons (Kempermann *et al.,* 1997; Kempermann *et al.,* 2002; Bruel‐Jungerman *et al.,* 2005) possibly mediated by the growth factor VEGF (vascular endothelial growth factor) (During and Cao, 2006), to enhance hippocampal synaptic strength, and to modify long‐term potentiation (LTP) (Duffy *et al.,* 2001; Foster and Dumas, 2001; Artola *et al.,* 2006). Furthermore, enrichment enhances levels of synaptic proteins (Frick and Fernandez, 2003; Nithianantharajah *et al.,* 2004) and brain neurotrophins [such as BDNF (brain‐derived neurotrophic factor) and NGF (nerve growth factor)] (Pham *et al.,* 1999; Ickes *et al.,* 2000) in the hippocampus and other brain regions, and modulates expression of genes linked to neuronal structure and synaptic transmission (Rampon *et al.,* 2000; Wassouf *et al.,* 2018). Moreover, exposure to environmental enrichment augments cortical and hippocampal dendritic branching and synaptogenesis (van Praag *et al.,* 2000). Similarly, enhanced physical activity increases cellular and synaptic plasticity by modulating synaptic structure and strength (Farmer *et al.,* 2004; Eadie *et al.,* 2005), elevating synaptic protein levels (Farmer *et al.,* 2004; Vaynman *et al.,* 2006), and promoting neurogenesis (van Praag *et al.,* 1999; Trejo *et al.,* 2001; Fabel *et al.,* 2003). An increase in dendritic length and complexity has also been linked to enhanced physical activity (Eadie *et al.,* 2005). Consistent with effects of EE, physical activity alone induces growth factors of several classes, including BDNF, IGF‐1 (insulin‐like growth factor‐1), and VEGF, that work in concert and through downstream signaling cascades to mediate the effects of the environment on the brain (Trejo *et al.,* 2001; Berchtold *et al.,* 2005; Kuipers and Bramham, 2006; Ding *et al.,* 2006a; Ding *et al.,* 2006b). Given their broad influence, it is plausible that such growth factors orchestrate the wide‐ranging impact of exercise and EE on brain functions and resilience (Cotman *et al.,* 2007). In this context, the enhancement in growth factor function induced by the environment can be due to either increasing growth factor levels itself or indirectly by improving the immune condition of the brain (Cotman *et al.,* 2007) as proinflammatory cytokines are able to impair growth factor signaling (Venters *et al.,* 2001; Tong *et al.,* 2008). Consequently, such morphological and molecular changes in response to EE and physical activity elicit behavioral improvement in learning and memory (Duffy *et al.,* 2001; Tang *et al.,* 2001; Vaynman *et al.,* 2004; van Praag *et al.,* 2005), neuropsychiatric behavior (Blumenthal *et al.,* 1999; Roy *et al.,* 2001; Benaroya‐Milshtein *et al.,* 2004; Singh *et al.,* 2005), and protect from age‐related and disease‐related neurodegeneration (Colcombe and Kramer, 2003; Weuve *et al.,* 2004; Nithianantharajah and Hannan, 2011; Hentrich *et al.,* 2018). The benefits of such environmental stimuli can be passed to the next generation as shown recently in mice (Benito *et al.,* 2018). As as intergenerational effect, the exposure to EE enhances hippocampal synaptic plasticity and improves cognitive abilities mediated through miR212/132 (Benito *et al.,* 2018).

## Environmental enrichment and brain degeneration

After the first evidence that environmental enrichment delays disease onset in a preclinical model of a brain disorder, extensive evidence on the benefits of EE in the context of neurodegenerative disorders has been rapidly accumulating (Nithianantharajah and Hannan, 2006; Wassouf *et al.,* 2018). The global impact of EE on brain health, such as stimulation of neurotrophic factors, immune system improvements, and neurogenesis, induces changes in brain functions and strengthens neuronal and synaptic connectivity, which may provide compensatory capacities and facilitate neuroplasticity that decelerates pathogenetic processes associated with brain neurodegeneration, a theory referred to as ‘brain reserve’ or ‘cognitive reserve’ (Stern, 2002; Valenzuela and Sachdev, 2006; Nithianantharajah and Hannan, 2009). In humans, the association between physical activity and a reduced risk for dementia and Alzheimer’s disease (AD) is evident in several clinical studies (Hamer and Chida, 2009; Brown *et al.,* 2013), and have also demonstrated a role for physical activity protecting from cognitive decline and maintaining a superior cognitive functioning in verbal memory, attention, executive functions, and general cognitive capabilities. Studies in AD mice, moreover, have shown broad behavioral and cellular alterations induced by EE (Cracchiolo *et al.,* 2007; Mirochnic *et al.,* 2009), including enhanced learning and memory (Arendash *et al.,* 2004), marked reduction in cerebral Aß levels and amyloid deposits, and increased neprilysin activity (Lazarov *et al.,* 2005).

As mentioned above, recent meta‐analyses have highlighted physical activity to have one of the most significant associations with a reduced risk of developing PD (Yang *et al.,* 2015; Bellou *et al.,* 2016). Specifically, a medium level of daily physical activity seems inversely associated with PD risk. Likewise, for PD patients, exercise exerts beneficial influences on quality of life and physical competencies, including leg strength, balance, and gait (Goodwin *et al.,* 2008), in line with known benefits of physiotherapy in PD patients (Keus *et al.,* 2007). Equally, treadmill exercise improves gait and postural stability in PD patients with mild to moderate impairments (Herman *et al.,* 2007; Herman *et al.,* 2009).

## Environmental enrichment in PD models

The impact of EE in PD was initially shown (Bezard *et al.,* 2003; Tillerson *et al.,* 2003; Faherty *et al.,* 2005; Jadavji *et al.,* 2006) and further followed‐up (Anastasia *et al.,* 2009; Goldberg *et al.,* 2011; Klaissle *et al.,* 2012; Jungling *et al.,* 2017) in toxin‐induced rodent models after neurotoxin insults with MPTP or 6‐hydroxydopamine (6‐OHDA), which cause damage of nigrostriatal dopaminergic neurons and depletion of striatal dopamine. Specifically, EE exposure in these models increases resistance to MPTP‐induced neuronal insult (Bezard *et al.,* 2003; Faherty *et al.,* 2005), and protects from or ameliorates behavioral impairments, including Parkinson’s related motor deficits in gait and balance, as a result of 6‐OHDA or MPTP insult (Tillerson *et al.,* 2003; Jadavji *et al.,* 2006; Pothakos *et al.,* 2009; Goldberg *et al.,* 2011). Preservation of dopaminergic neurons, dopaminergic fibers in the striatum, and dopamine transporters, decreased loss of striatal dopamine along with enhanced neurotrophic factors such as BDNF and glial cell‐derived neurotrophic factor (GDNF) have been further associated with EE exposure in these models (Bezard *et al.,* 2003; Cohen *et al.,* 2003; Tillerson *et al.,* 2003; Faherty *et al.,* 2005; Tajiri *et al.,* 2010). Moreover, physical exercise increases dopamine production in remaining dopaminergic neurons in PD (Sutoo and Akiyama, 2003), and modifies glutamate signaling, either by influencing expression of glutamate receptors or the presynaptic storage and release of glutamate (VanLeeuwen *et al.,* 2010). The beneficial impact of EE might also involve the immune system, counteracting the activation of the immune response observed in the context of PD pathology (Huang and Halliday, 2012; Heneka *et al.,* 2014). Notably, an alternative mechanism for the EE‐induced neuroprotection in the toxin‐models of PD may be through lowering dopamine transporter (DAT) levels, which is required for 6‐OHDA and MPTP uptake and neurotoxicity (O'Dell *et al.,* 2007; Petzinger *et al.,* 2007).

To better understand these principles, it is essential to extend these investigations to genetic animal models of PD. While several genetic PD models, for example for *SNCA,* are available, reports on EE impact in such models of familial PD forms are still scarce. Recently, a study by Minakaki and colleagues has shown benefits of physical exercise in human alpha‐synuclein expressing mice with motor enhancement, particularly in gait and posture, and augmented striatal tyrosine hydroxylase levels after treadmill exercise (Minakaki *et al.,* 2019). Further, EE exposure improves olfactory function and alleviates oxidative stress and levels of nitrated α‐syn in human A53T α‐syn overexpressing mice (Wi *et al.,* 2018). In a recent study from our lab, we investigated the influences of a long‐term EE in a mouse model overexpressing the human wildtype *SNCA* gene under its native regulatory elements to trace molecular events that connect EE influences to α‐syn biology (Wassouf *et al.,* 2018). Environmental enrichment led to widespread prevention of *SNCA*‐induced disturbances in the hippocampal transcriptome including disturbances in microglia and astrocytes. These preventive effects were accompanied by sustained activation of a group of immediate early genes (IEGs), including the transcription factors EGR1 and NURR1/NR4A2 (Wassouf *et al.,* 2018). Such approaches have the potential to highlight intriguing regulatory networks that might harbor attractive therapeutic points of action to mimic beneficial impacts of EE and open novel avenues for preventing, delaying, or treating synucleopathies and related disorders early on.

## Environmental stress and brain health

Our brain is constantly changing with experience and the discussed EE effects on brain health and disease are examples for the brain’s plasticity and adaptability. The brain’s adaptation to the surrounding environment further involves perceiving and responding to environmental stressors (McEwen *et al.,* 2015). While healthy brains are capable of coping and adapting to stressors, an individual resilience or vulnerability to stress can be shaped by several factors, such as type, duration, and intensity of the stressor, the individual’s gender, genetic and epigenetic background, as well as early life experiences (McEwen, 2007; Sotiropoulos *et al.,* 2008; McEwen and Morrison, 2013; Bale and Epperson, 2015; Chen and Baram, 2016; Sousa, 2016). Further, stress response can be impaired by aging processes or neurodegenerative diseases and render individuals susceptible to detrimental physical and mental consequences (Hatzinger *et al.,* 1995; Hartmann *et al.,* 1997; Charlett *et al.,* 1998; Peskind *et al.,* 2001; Aguilera, 2011). Regulating stress response in the brain involves hypothalamic and extra‐hypothalamic structures that express glucocorticoid receptor (GR) alone or together with mineralocorticoid receptor. Both receptors respond to glucocorticoids (GC) with varying affinity and mediate GCs’ broad effects in the brain (Veldhuis *et al.,* 1982; McEwen *et al.,* 1986), which may involve neuronal and glial cells (Ahima *et al.,* 1991). Because of higher mineralocorticoid receptor affinity for GC (10 fold higher), these receptors are occupied at basal GC levels, while GR is activated only at a certain GC threshold (Reul and de Kloet, 1985; De Kloet *et al.,* 1998).

## Environmental stress and neurodegeneration

**Figure 2:**
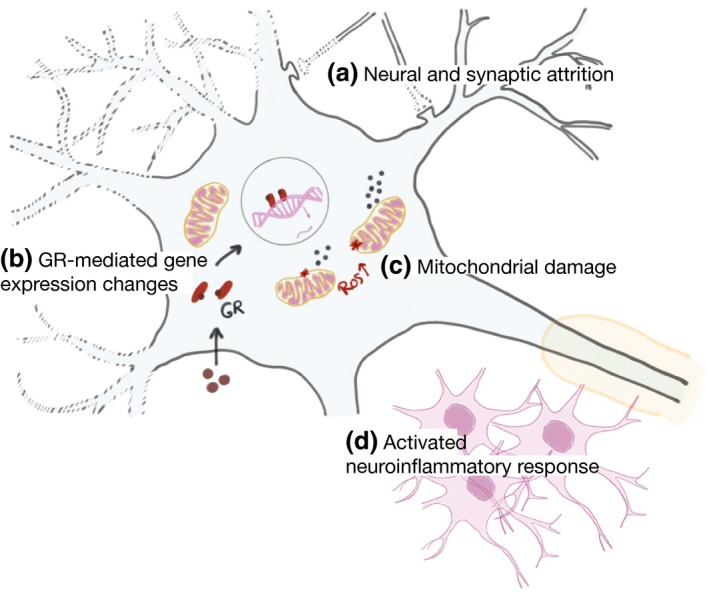
Pathological consequences of environmental stress and elevated stress hormones on the brain. (a) Neural and synaptic attrition. (b) Glucocorticoids (GCs) are released as a result of stress and their actions are mediated by binding to glucocorticoid receptors (GRs), GRs act as transcription factors causing changes in gene expression. (c) Mitochondrial damage and increase of ROS production. (d) Activated neuroinflammatory response. ROS: Reactive oxygen species.

Several lines of evidence propose a link between prolonged stress exposure causing elevated stress hormones (GCs) and certain brain disorders including occurrence and progression of neurodegenerative diseases (Mejia *et al.,* 2003; Smith *et al.,* 2008; Simard *et al.,* 2009; de Pablos *et al.,* 2014; Di Meco *et al.,* 2014). The pathological consequences of prolonged stress and elevated stress hormones on the brain comprise altering a wide range of neuronal (and non‐neuronal) functions and structures (McEwen *et al.,* 2015; Vyas *et al.,* 2016) through mechanisms that involve transcriptomic and epigenetic modulations, among others (Meaney and Szyf, 2005; Gray *et al.,* 2014; Hunter *et al.,* 2015). These effects are mediated by glucocorticoid receptors acting as transcription factors together with other interacting cellular mediators. The resulting neuronal attrition and brain pathology due to stress/GC elevation resembles those of neurodegenerative disorders, like distorted neuronal architecture, synaptic damage, mitochondrial dysfunction, altered neurogenesis, and activated neuroinflammatory response (McEwen *et al.,* 2015; Vyas *et al.,* 2016) (Fig. 2). In fact, clinical data and experimental studies exploring the role of stress and GCs in neurodegenerative diseases such as PD, AD, and dementia further support these commonalities (Smith *et al.,* 2002; Mejia *et al.,* 2003; Metz, 2007; Pienaar *et al.,* 2008; Simard *et al.,* 2009; Zou *et al.,* 2013).

## Environmental stress in PD models

Combining stress exposure with MPTP or 6‐OHDA insults in toxin‐induced animal models of PD exacerbates neuronal damage, aggravates motor deficits, and triggers neuroinflammatory response in rodents (Pienaar *et al.,* 2008; Smith *et al.,* 2008; Lauretti *et al.,* 2016). These findings and several others (Mizoguchi *et al.,* 2000; Rasheed *et al.,* 2010; de Pablos *et al.,* 2014) have unveiled an overt susceptibility of the dopaminergic circuitries to stress‐induced cytotoxicity. Such vulnerability of the dopaminergic system can be related to prolonged release of catecholamines such as dopamine, GCs, and glutamate as a result of chronic stress (Smith *et al.,* 2002). Particularly, catecholamines, including dopamine, are prone to auto‐oxidation, which can ultimately lead to neurodegeneration (Goldstein, 2011). In fact, mild chronic stress has already been reported to trigger oxidative stress (Lucca *et al.,* 2009). Chronic stress, moreover, induces a proinflammatory response and release of cytokines and chemokines that result in activation of the hypothalamic‐pituitary‐adrenal (HPA) axis (Haddad *et al.,* 2002). On a related note, changes in GR have been described in PD patients and MPTP‐intoxicated models, and sustained proinflammatory response resulting in dopaminergic neurons degeneration has been observed after specific modulation of the microglial GR in MPTP‐treated mice (Ros‐Bernal *et al.,* 2011). Altogether, stress and sustained elevation of GCs potentially impact motor as well as non‐motor features of PD such as anxiety and depression already surfacing during PD’s prodromal phase (Goldman and Postuma, 2014; Bellou *et al.,* 2016). Some PD characteristic traits associated with anxiety seemingly result from damage to the mesolimbic dopaminergic projections involved in reward and motivation, such as lower novelty seeking and higher harm avoidance (Menza *et al.,* 1993; Kaasinen *et al.,* 2001; Costa and Caltagirone, 2015).

Similar to studies on environmental enrichment, the majority of reports examining the impact of environmental stress in PD models focus on the effects on dopaminergic neurons and dopamine depletion triggered by neurotoxins in toxin‐induced models. Whether environmental stressors have the same impact in genetic models of PD is still poorly investigated. In a study by Wu and colleagues, chronic stress has been shown to trigger motor impairment and degeneration of dopaminergic neurons accompanied by an increase of abnormal α‐syn inclusions and activation of a proinflammatory response in A53T mice (Wu *et al.,* 2016). In the same genetic model, chronic restrain stress accelerates motor deficits and aggravates pathological signs linked to PD, possibly via the activation of RTP801, also known as the stress‐responsive gene DNA damage‐inducible transcript 4 (DDIT4) (Zhang *et al.,* 2018). Inhibition of RTP801 alleviates neurodegeneration and PD‐like symptoms in stress‐treated A53T mice (Zhang *et al.,* 2018). Further, higher anxiety‐related behavior and abnormal response to immobilization stress have been described in A53T mice, which were accompanied by aberrant regulation of dopamine β‐hydroxylase (Kim *et al.,* 2014). Another study from our lab has investigated the effects of chronic unpredictable stress exposure on a genetic α‐syn model (Wassouf *et al.,* 2019). In this work, we provide evidence on an altered stress response in *SNCA*‐overexpressing mice complemented by aberrant anxiety‐related behavior. These phenotypic manifestations precedes the presence of motor impairments and concurres with modulations of the striatal transcriptome affecting genes linked to neuroinflammation, synaptic signaling, and neurotransmission in *SNCA*‐overexpressing mice. Importantly, further research that delves into exploring the molecular transducers of the environmental influences will aid shedding light on the intriguing interplay of environment and *SNCA* related processes.

## Conclusion and outlook

While evidence is converging on the impact of a variety of environmental influences on cause and course of neurodegenerative disorders, there seems to be no single universal environmental factor triggering these disorders. Instead, a combination of environmental elements, genetic predispositions, and aging processes seemingly prime the demise of a specific subset of neurons in these diseases. Identifying the molecular events that govern such demise and that mediate the impact of each of these interacting factors may shed light on potential molecular targets to reverse a physiological state to ultimately prevent, attenuate, or cure these diseases. Because of the dynamic nature of the transcriptome and the epigenome in response to environmental stimuli, these regulatory layers may harbor attractive targets for molecular modulation that eventually exerts its effects on functional and phenotypic endpoints. Hence, several research projects have been and further should be geared towards identifying such molecular targets that either mimic or oppose the environmental or disease attributes. Importantly, translating these findings to clinical trials entails conducting animal studies with high relevance to the human conditions as discussed before (Nithianantharajah and Hannan, 2006). In this context, a valid question is raised, namely whether standard housing for rodents with typically low levels of environmental stimulation is an appropriate control housing or rather a deprived condition compared to environmental enrichment which may better mimic our daily complex and stimulating lives. The observed beneficial effects of EE, in this case, may only be due to worsening of disease‐related phenotypes in animals housed in deprived standard‐housing conditions. Yet, the observed effects of EE remain relevant corroborated by reports employing higher levels of enrichment referred to ‘super enrichment’ (Mazarakis *et al.,* 2014). Accounting for such considerations when interpreting findings from animal studies is vital to facilitate translation to clinical practice and define the type of environmental intervention necessary to achieve the functional benefits we aim for in patients.
